# High-throughput deep sequencing reveals the important role that microRNAs play in the salt response in sweet potato (*Ipomoea batatas* L.)

**DOI:** 10.1186/s12864-020-6567-3

**Published:** 2020-02-17

**Authors:** Zhengmei Yang, Panpan Zhu, Hunseung Kang, Lin Liu, Qinghe Cao, Jian Sun, Tingting Dong, Mingku Zhu, Zongyun Li, Tao Xu

**Affiliations:** 1 Key Lab of Phylogeny and Comparative Genomics of the Jiangsu Province, Institute of Integrative Plant Biology, School of Life Sciences, Jiangsu Normal University, Xuzhou, 221116 Jiangsu Province China; 2 Department of Plant Biotechnology, College of Agriculture and Life Sciences, Chonnam National University, Gwangju, 500-757 South Korea; 3 Guangdong Provincial Key Laboratory for Plant Epigenetics, Longhua Bioindustry and Innovation Research Institute, College of Life Sciences and Oceanography, Shenzhen University, Shenzhen, 518060 Guangdong China; 4 Xuzhou Academy of Agricultural Sciences/Sweet Potato Research Institute, CAAS, Xuzhou, 221121 Jiangsu China

**Keywords:** Sweet potato, Salt stress, microRNA, High-throughput sequencing, Degradome sequencing

## Abstract

**Background:**

MicroRNAs (miRNAs), a class of small regulatory RNAs, have been proven to play important roles in plant growth, development and stress responses. Sweet potato (*Ipomoea batatas* L.) is an important food and industrial crop that ranks seventh in staple food production. However, the regulatory mechanism of miRNA-mediated abiotic stress response in sweet potato remains unclear.

**Results:**

In this study, we employed deep sequencing to identify both conserved and novel miRNAs from salinity-exposed sweet potato cultivars and its untreated control. Twelve small non-coding RNA libraries from NaCl-free (CK) and NaCl-treated (Na150) sweet potato leaves and roots were constructed for salt-responsive miRNA identification in sweet potatoes. A total of 475 known miRNAs (belonging to 66 miRNA families) and 175 novel miRNAs were identified. Among them, 51 (22 known miRNAs and 29 novel miRNAs) were significantly up-regulated and 76 (61 known miRNAs and 15 novel miRNAs) were significantly down-regulated by salinity stress in sweet potato leaves; 13 (12 known miRNAs and 1 novel miRNAs) were significantly up-regulated and 9 (7 known miRNAs and 2 novel miRNAs) were significantly down-regulated in sweet potato roots. Furthermore, 636 target genes of 314 miRNAs were validated by degradome sequencing. Deep sequencing results confirmed by qRT-PCR experiments indicated that the expression of most miRNAs exhibit a negative correlation with the expression of their targets under salt stress.

**Conclusions:**

This study provides insights into the regulatory mechanism of miRNA-mediated salt response and molecular breeding of sweet potatoes though miRNA manipulation.

## Background

Soil salinity, one of the major environmental factors, reduces the productivity of crops worldwide [1]. It is estimated that 50% of all arable land will be affected by salinity by 2050 [2], and approximately 20% of the irrigated soils worldwide are suffering salt stress [3]. Meanwhile, the crop production demands continue to increase with the rapidly growing population [4]. Therefore, to sustain or increase food supply, salt tolerance is an important agronomic trait to support crop plant growth and production in marginal and high-salinity soils [5].

MicroRNAs (miRNAs) are a class of endogenous small non-coding RNAs (sRNAs) that are 21–24 nt in length and negatively regulate gene expression at transcriptional and post-transcriptional levels [6–8]. In plants, the primary miRNA precursor is transcribed from DNA and then sequentially processed by Dicer-like 1 (DCL1) via two steps: firstly, into precursor-miRNAs (pre-miRNAs), and secondly, into miRNA/miRNA* duplex. The mature miRNA from one of the duplex strands incorporates into the RNA-induced silencing complex (RISC) and then guides the RISC to target mRNA, either cleaving the target with near perfect base pairing complementarity or repressing its translation with lower complementarity [9, 10]. Previous studies showed that miRNAs regulate diverse processes in plants, including leaf morphogenesis and polarity, root initiation and development, flower differentiation and development, stem and vascular development, phase switch from vegetative growth to reproductive growth and response to abiotic and biotic stresses [7, 11, 12].

Salinity stress affects the photosynthesis, signal transduction, protein synthesis and degradation processes in plants and inhibits crop yield dramatically. miRNAs are indispensable for plants to respond to salt stress and play an important regulatory role by regulating the expression of their target genes. For example, miR396 increases *Arabidopsis* salt tolerance through regulating its target gene *GRF* [13]; miR398 protects cell membrane structure against salt stress by targeting *CSD1* and *CSD2* [14]; The miR172c-NNC1 module modulates root plastic development in response to salt in soybean [15]; and the target gene of miR169c can control the stomatal opening and closure in maize leaves, thereby reducing water loss and resisting salt stress [16]. The decrease in miR414/miR408/miR164e leads to up-regulated helicase (*OsABP*, *OsDSHCT* and *OsDBH*) expression, thereby regulating gene recombination replication and repair to resist saline environment [17]. The overexpression of osa-miR393 negatively regulates rice salt tolerance [18]. The miR397-resistant transgenic *Arabidopsis* plant decreases its salt tolerance ability [19]. MiR319 and miR528 can enhance cotton plant tolerance to salt stress by down-regulating their target genes [20, 21]. Recently, deep sequencing data showed that several miRNAs are significantly altered under NaCl stress in various plants, such as rice [22], soybean [23] and *Dunaliella salina* [24]. Evidence suggests that miRNA plays an important regulatory role in plant salt stress tolerance.

Sweet potato (*Ipomoea batatas* L.) is an important food and industrial crop [25]. It is a valuable food source that contains various nutrients, including high starch content, complex carbohydrates, dietary fibre, vitamins and anthocyanins. Salt stress adversely influences sweet potato growth, fresh weight, health-promoting compounds and antioxidant activity. As a source of bio-energy, sweet potato is mainly planted on marginal land; hence, improving its salt tolerance is important to maintain productivity [26, 27]. Until now, a series of salt-tolerance-associated genes, such as *IbOr*, *IbNFU1*, *IbP5CR*, *IbMas*, *IbSIMT1*, *IbMIPS1* and *IbZFP1*, has already been well characterised in sweet potatoes [26, 28–35]. However, very little work has been done on sweet potato miRNAs, and miRNA-related salt stress in sweet potato has not been investigated.

In the present study, we employed high-throughput sequencing technology and bioinformatic analysis to identify conserved and novel salt-responsive miRNAs by constructing small RNA libraries from NaCl-free (CK) and NaCl-treated (Na150) sweet potato roots and leaves. The expression profiles of the miRNAs between the roots and the leaves were investigated. We also predicted the targets of miRNAs and further investigated their network by GO and KEGG analyses using sweet potato transcriptome and degradome data. This study contributed in elucidating the potential miRNA-mediated regulatory mechanism of salt stress response in sweet potatoes. The specific miRNAs in sweet potatoes can be used to breed salt-tolerant plants that can grow on marginal lands.

## Results

### Transcriptome sequencing analysis

The transcriptome sequencing of sweet potato (Xu32) was performed by Illumina high-throughput sequencing. Approximately 90,528,282, 109,002,500, 82,914,414 and 90,852,454 raw reads were obtained from the SRC, SLC, SRN and SLN libraries, respectively, and the total number of bases was approximately 56.01Gb (Additional file 1: Table S1). Through the Trinity de novo assembly method, a total of 27,712 non-redundant genes and 41,879 transcripts were obtained. The N50 length of these transcripts was 1338 bp, with an average length of 924 bp and a total of 25,967 transcripts over 500 bp (63%) (Additional file 2: Table S2 and Additional file 3: Table S3). All unigenes were searched in the Swiss-Prot, Nr, Pfam, KEGG, KOG and GO databases, and a total of 14,671, 22,756, 17,289, 8516, 19,462 and 12,959 were detected, respectively (Additional file 4: Table S4).

### Sequencing, annotation and sequence characterisation of small RNA

**Fig. 1:**
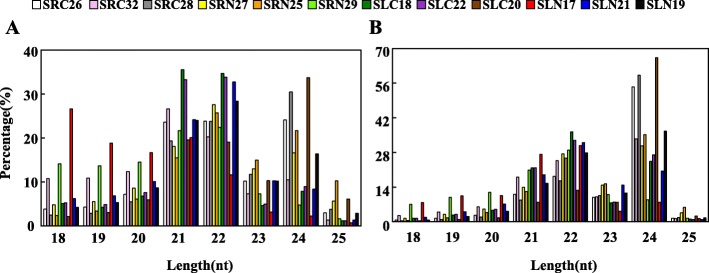
Length distribution and abundance of small RNAs in 12 sRNA libraries in sweet potato. **a** Size distribution of redundant sequences. **b** Size distribution of unique sequences

To explore the regulatory mechanisms of miRNAs in response to salt stress in sweet potato, we established four different sample groups, namely, NaCl-treated (Na150) group of roots (SRN) and leaves (SLN) and the NaCl-free group of roots (SRC) and leaves (SLC). Every sample group contains three biological replicates. As listed in Additional file 5: Table S5, a total of 12 sRNA libraries were established and subjected to high-throughput sequencing. Using Illumina’s Solexa sequencing platform, raw data were obtained as follows: 16,855,549 (SLC26), 20,085,489 (SLC28), 19,050,745 (SLC32), 17,881,455 (SLN18), 21,223,113 (SLN20), 17,902,270 (SLN22), 20,477,313 (SRC25), 20,464,252 (SRC27), 17,965,687 (SRC29), 16,221,626 (SRN17), 18,904,028 (SRN19) and 22,740,499 (SRN21). After filtering low-quality data and 3′ joint contamination data and sequences with length less than 18 nt or greater than 25 nt and excluding non-coding RNAs (e.g. rRNAs, tRNAs, snRNAs and snoRNAs), the remaining unannotated data were used for miRNA identification. The size distributions of the 12 small RNA libraries are presented in Fig. 1. The distribution patterns of the most redundant sRNA peaks at 21, 22 and 24 nt are shown in Fig. 1a. However, in the distribution pattern of non-redundant sRNA length, 24 nt sRNAs are the most abundant category (average of 33.7%) followed by 22 and 21 nt (Fig. 1b).

### Identification of known miRNAs in sweet potato

**Fig. 2:**
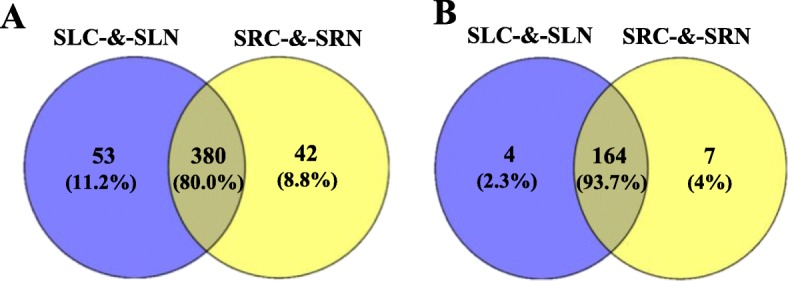
Distribution of miRNAs in sweet potato roots and leaves. **a** Distribution of known miRNAs in sweet potato roots and leaves. **b** Distribution of novel miRNAs in sweet potato roots and leaves

**Fig. 3:**
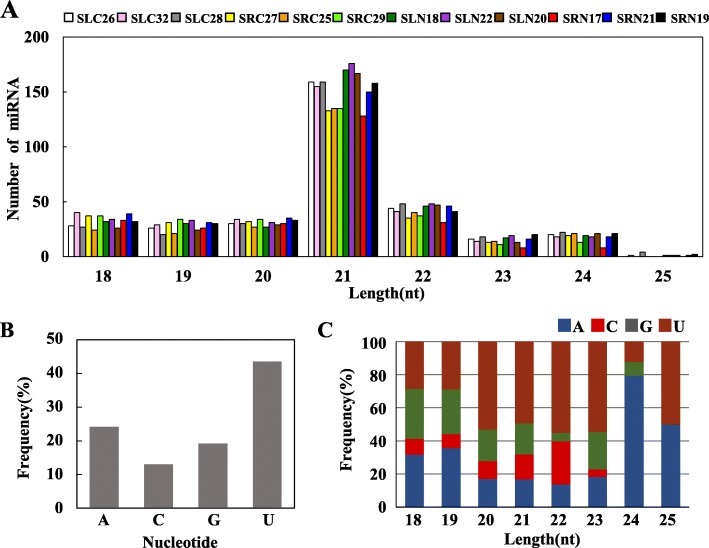
Analyses of the length distribution and nucleotide base bias of known miRNAs. **a** Size distribution of known miRNA. **b** Percentage of first nucleotide bias in known miRNAs. **c** Known miRNA nucleotide bias at first position

**Fig. 4:**
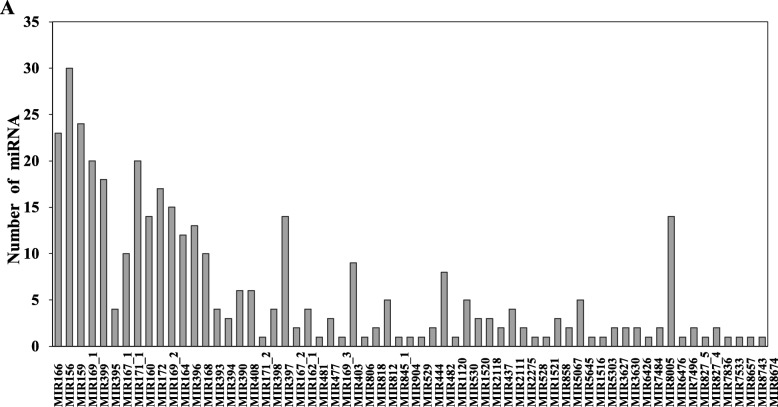
Distribution of conserved miRNAs in miRNA family

To identify the known miRNAs in sweet potato, we screened the sequences that matched the sweet potato transcriptome sequences in the 12 small RNA libraries and then aligned these sequences with the matured miRNA sequences in miRBase 21.0 database. Finally, 444 pre-miRNAs corresponding to 475 known unique mature miRNAs were identified as homologues of known miRNAs from the other 42 plants (Additional file 6: Table S6). Approximately 33.89% of these sweet potato miRNAs can be found in Solanaceae plants, such as *Solanum tuberosum* (111), *Solanum lycopersicum* (19) and *Nicotiana tabacum* (31) (Additional file 7: Figure S1). Among these known miRNAs, 53 and 42 were specifically expressed in sweet potato leaves and roots, respectively, and 380 were co-expressed in the roots and leaves (Fig. 2a). The length of known miRNAs ranges from 18 nt to 25 nt, with 21 nt miRNAs being the most abundant (41.47%) (Fig. 3a). In the first nucleotide selection, uracil accounted for 53.19, 49.49, 55.17 and 54.55% in miRNAs of 20 nt to 23 nt in length, respectively (Fig. 3b and Fig. 3c). Among these known miRNAs, 379 belong to 66 families (Additional file 6: Table S6 and Fig. 4), whereas the families of the other 96 miRNAs are unknown. The three largest families were miR156 (30 miRNA members), miRNA159 (24 members) and miR166 (23 members), whereas only 22 families contained only 1 member (Fig. 4). Among these 66 miRNA families, 55, 58, 54 and 49 families were expressed in the SRN, SRC, SLN and SLC library, respectively (Additional file 8: Figure S2).

High-throughput sequencing detects the type of miRNA and the abundance of different miRNAs [36]. The expression abundance of some miRNA families in the four libraries varied (Additional file 8: Figure S2). Eleven families (miR166, miR156, miR168, miR2111, miR2275, miR398_2, miR858, miR6426, miR827_5, miR827_4 and miR8674) were up-regulated in the roots and leaves of sweet potato under salt stress condition, and 15 families (miR169_1, miR164, miR390, miR408, miR397, etc.) were down-regulated (Additional file 8: Figure S2). A total of 24 miRNA families exhibited opposite expression patterns in sweet potato leaves and roots under salt stress condition. For example, miR162_1 and miR398 were down-regulated in the roots but up-regulated in the leaves, whereas miRNA159 and miRNA160 were up-regulated in the roots but down-regulated in the leaves. miR166, miR168, miR2118, miR397, miR156, miR398, miR396, miR167_1, miR159 and miR408 were highly expressed in all four libraries. The different abundance of miRNAs indicated that they played different roles under salt stress.

### Identification of novel miRNAs in sweet potato

According to the annotation standards of novel miRNA [37] and after normalising the expression level, miRNAs with a copy number of less than 10 were eliminated in all samples. Finally, 175 novel miRNAs were predicted from 157 pre-miRNAs with a length of 21–24 nt (Additional file 9: Table S7 and Additional file 10: Figure S3A), which was in line with the size of miRNA fragments generated by AGO1 protein cleavage. Among these novel miRNAs, 4 were specifically expressed in the leaves, 7 were specifically expressed in the roots, and 163 were co-expressed in the roots and leaves (Fig. 2b). Among the novel miRNAs, the first nucleotide of 5′ was A (adenine) (33.91%) and U (uracil) (35.63%) (Additional file 10: Figure S3B and S3C). These pre-miRNAs range in length from 59 nt to 259 nt with an average length of 138 nt, which is consistent with the general length of pre-miRNAs. The CG percentages (CG%) of these novel pre-miRNAs range from 12.20 to 61.20%, and their minimal folding free energy index (MFEI) ranges from 0.90 to 2.50 with an average of 1.29. The secondary structure of the precursor of the novel miRNA was obtained using Mfold software, and the structure of the representative miRNA is listed in Additional file 11: Figure S4.

### Differential expression analysis of miRNAs in sweet potato under salt stress condition

**Fig. 5:**
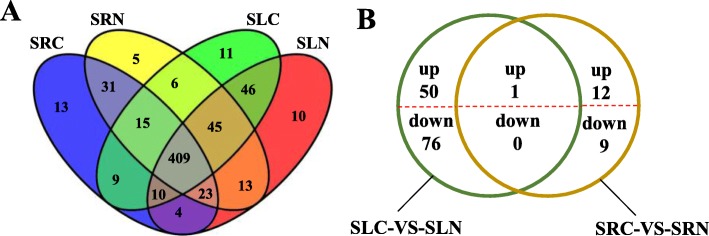
Distribution of miRNAs in CK and NaCl (150 mM) groups in sweet potato roots and leaves. **a** Distribution of total miRNAs in CK and NaCl (150 mM) groups in sweet potato roots and leaves. **b** Differentially expressed miRNAs in sweet potato roots and leaves were identified through two comparisons (SRC-VS-SRN and SLC-VS-SLN). MiRNAs with significant differences in sweet potato roots and leaves are shown in Additional file 12: Table S8

To find the salt-stress-responsive miRNAs in sweet potato (Xu32), the expression levels of all miRNAs were normalised and analysed. As shown in Fig. 5a, 33 and 28 miRNAs were specifically expressed in the control and under the salt stress condition, respectively. In the leaves, 41 and 50 miRNAs were specifically expressed in NaCl-free (SLC) and NaCl (150 mM) (SLN) groups, respectively; in the roots, 36 and 69 miRNAs were specifically expressed in CK (SRC) and NaCl (150 mM) (SRN) groups, respectively. In addition, 10, 11, 5 and 13 miRNAs were specifically expressed in SLN, SLC, SRN and SRC libraries, respectively (Fig. 5a). These results implied that the specifically expressed miRNAs under the control condition may play a negative role in salt response, whereas the specifically expressed miRNAs under salt condition may play a positive role in the salt response in sweet potato roots and/or leaves.

A total of 148 miRNAs (101 known miRNAs and 47 novel miRNAs) were significantly (*P* < 0.05 and |log2(fold change)| > 1) up- or down-regulated in sweet potato under salt stress (Additional file 12: Table S8, Fig. 5b). In the leaves, 50 miRNAs (21 known miRNAs and 29 novel miRNAs) were up-regulated, whereas 76 miRNAs (61 known miRNAs and 15 novel miRNAs) were down-regulated; in the roots, 12 miRNAs (11 known miRNAs and 1 novel miRNA) were up-regulated, whereas 9 miRNAs (7 known miRNAs and 2 novel miRNAs) were down-regulated. Among all the differentially expressed miRNAs, only one miRNA (ath-mir164b-3p) was up-regulated both in the roots and leaves after salt treatment. Among these significant differentially expressed miRNAs in leaves, six miRNAs (nta-miR156a_R + 3, far-miR159_L + 2_1ss22T, mes-MIR319e-p5_2ss12GC19GA, ssl-miR171b_1ss21TA, nta-miR169q_1ss14CA, PC-3p-786500_26) were only detected in the SLC library, whereas four miRNAs (gma-miR156b_L + 2R-1, gma-MIR171a-p5_2ss12TA18AC, PC-3p-14376_1292, PC-5p-150993_155) were only detected in the SLN library (Additional file 12: Table S8). In the roots, lus-MIR169j-p3-2ss6TC21TG and tcc-miR530a_R + 1_1ss12CT were only detected in the SRN library (Additional file 12: Table S8), indicating that these miRNAs may function in a tissue-specific manner in sweet potato under salt stress.

### Target gene prediction and annotation of miRNA

**Fig. 6:**
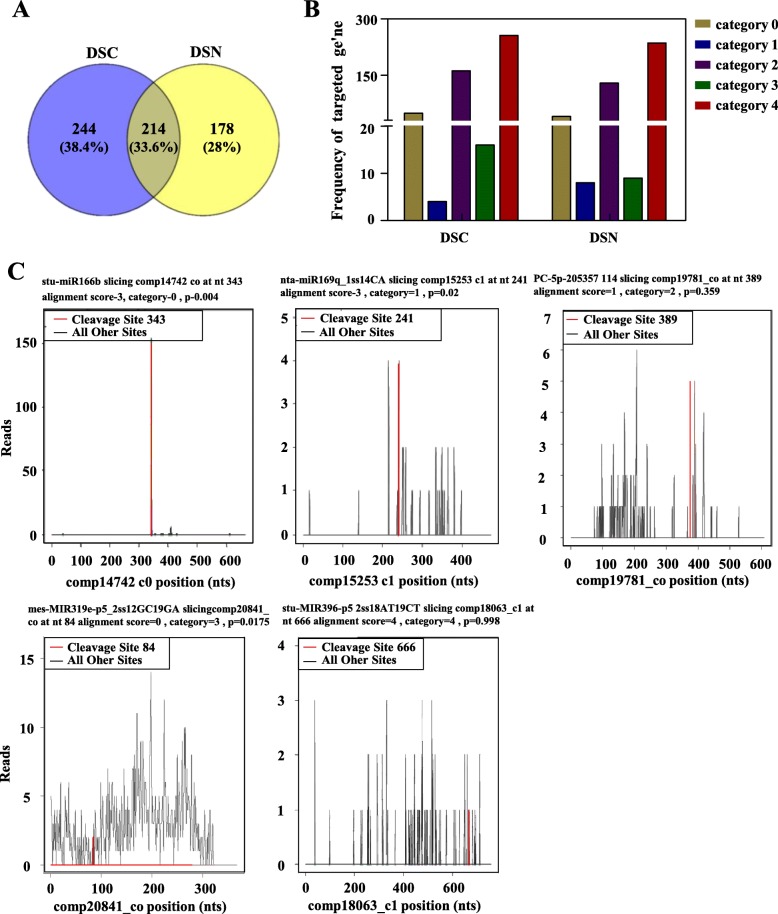
Target gene cleavage site analysis of miRNA. **a** Distribution of confirmed miRNA targets, separated by category in DSC library (left) and DSN library (right). **b** Distribution of target genes in two degradation group libraries. **c** Representative target plots (t-plot) depicting categories of the cleaved mRNAs confirmed by degradome sequencing, reflecting the reliability of the miRNA targets

To identify the target genes of miRNAs, we performed the miRNA-degradome of sweet potato and obtained 21,372,881 raw reads and 13,953,861 unique reads from the NaCl-treated (Na150) group (DSN) and 16,764,899 raw reads and 5,659,818 unique reads from the NaCl-free (CK) group (DSC) (Table 1). A total of 6,599,927 and 11,157,651 reads (called Transcript Mapped reads) mapped to the sweet potato transcriptome database were retained in DSN and DSC. Finally, 23,926 and 24,656 reads containing miRNA-mediated cleavage sites were identified in DSN and DSC. These data along with the sweet potato miRNA dataset were analysed according to the CleaveLand pipeline process [38]. Finally, the miRNA–mRNA pairings were obtained, that is, the target genes of the miRNA were identified. On the basis of the degradome data, a total of 392 target genes for 258 miRNAs (235 known miRNAs, 23 novel miRNAs) were identified in DSN (Additional file 13: Table S9 and Fig. 6a), and 458 target genes for 270 miRNAs (239 known miRNAs, 31 novel miRNAs) were identified in DSC (Additional file 13: Table S9 and Fig. 6a). Venn diagram analysis showed that 214 target genes were found in the two degradomes, and 244 and 178 specific target genes were present in the DSC and DSN libraries, respectively (Fig. 6a). According to the relationship between the cut position of the original fragment and the abundance of the mRNA, the target genes can be divided into five categories [39] (Additional file 14: Table S10). In the DSC and DSN libraries, the numbers of cleaved transcripts were 49 and 40 in Category 0; 4 and 8 in Category 1; 162 and 129 in Category 2; 16 and 9 in Category 3; and 256 and 236 in Category 4, respectively (Fig. 6b and Fig. 6c). In addition, on the basis of the sequencing results of the degradome, the same target gene corresponding to the different miRNAs may have different categories (Additional file 13: Table S9). For example, SPL13A (comp19729_c0), which is regulated by gma-mir156b_r-1, nta-mir156a_r + 1 and nta-mir156a_r + 3, belongs to Category 2 in the DSC library and Category 0 in the DSN library. In addition, conserved miRNAs can regulate multiple target genes. For example, ppe-MIR169i-p3_2ss1TC17GT regulates *TOP1*, *GC4*, *HSP90*, *UBXN1*, etc.; ath-miR156h_L + 1 regulates *SPL13A*, *SBP1* and SPL12. A target gene was regulated by multiple conserved miRNAs. For example, *AFB2* was co-regulated by stu-miR393-5p, stu-miR393-5p_R + 1_2ss21CT22CT and stu-miR393-5p_R-1_1ss21CT; *DCL1* was regulated by stu-miR162a-3p and stu-miR162a-3p_R-2.

**Table 1:** Summary of the degradome sequencing data in DSN and DSC

Sample	DSN		DSC	
	Number	Ratio	Number	Ratio
Raw reads	21,372,881	/	16,764,899	/
Reads <15 nt after removing 3 adaptor	73,472	0.34%	55,482	0.33%
Mappable reads	21,299,409	99.66%	16,709,417	99.67%
Unique raw reads	13,953,861	/	5,659,818	/
Unique reads <15 nt after removing 3 adaptor	54,807	0.39%	33,541	0.59%
Unique mappable reads	13,899,054	99.61%	5,626,277	99.41%
Transcript mapped reads	6,959,927	32.56%	11,157,651	66.55%
Unique rranscript mapped reads	2,095,096	15.01%	2,287,431	40.42%
Number of input transcript	27,712	/	27,712	/
Number of coverd transcript	23,926	86.34%	24,656	88.97%

### Functional analysis of the target genes of significantly up- or down-regulated miRNA under salt stress

Although 148 miRNAs were significantly reduced or induced by salt stress, 66 miRNAs and 87 target genes generated 168 regulatory interactions according to the combined results of miRNA profiling, degradome sequencing and transcriptome profiling. Among 87 target genes, 56 genes are transcription factors (TFs), such as *AP2*, *NAC*, *NFYA*, *GRF*, *SCL*, *SPL* and *TCP* (Additional file 15: Table S11 and Additional file 16: Table S12), which are involved in the regulation of gene expression and signal transduction. The other miRNA target genes, including desaturase, peptidase, synthase, transferase and kinase-encoding genes (such as calcium-dependent protein kinase (nta-miR169q_L + 2R-2, comp15536_c0), omega-3 fatty acid desaturase (mes-MIR319e-p5_2ss12GC19GA, comp23770_c0) and Cu^2+^-exporting ATPase (stu-miR408b-3p, comp11674_c0)) were involved in plant growth and developmental adaptive responses. Nine target genes of significantly up- or down- regulated novel miRNAs have been successfully identified. Among them, only four target genes were annotated, namely, formin-like protein 20, small subunit ribosomal protein S19, translation initiation factor if-3 subunit 6 and mitochondrial import receptor subunit TOM40. These results indicated that miRNAs may play an important role in different biological processes under salt stress in sweet potato.

**Fig. 7:**
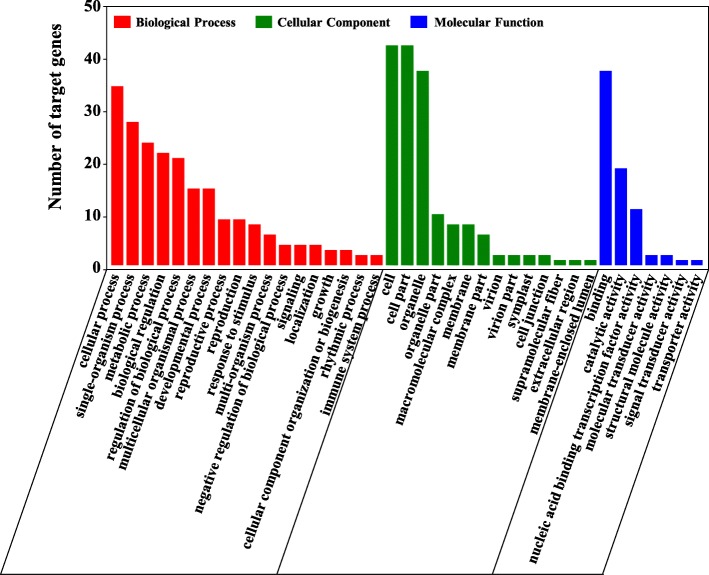
GO analysis of significant differentially expressed miRNA target genes in response to salt stress. The y-axis represents the percent of a specific category of target genes in that main category. The x-axis represents the name of the GO sub-categories

To further analyse the specific biological functions of sweet potato miRNAs under salt stress, we performed GO enrichment analysis on 87 target genes of 66 miRNAs in the regulatory interactions. The results indicated that the target genes were annotated into 39 GO terms (Fig. 7). Among them, the target genes of differentially expressed miRNAs have the largest proportion of cellular processes, followed by single-organism processes, metabolic process, biological regulation and regulation of biological processes. In the classification of cellular components, cells, cell parts and organelles (plasma membrane) have the top three number of genes (43, 43 and 38, respectively); in the molecular function classification, the two GO terms of binding and catalytic activity have the most target genes. In addition, we performed a significant analysis of these GO terms (*P* < 0.05, FDR < 0.05) (Additional file 17: Table S13). For the molecular function of target genes, the nucleic acid binding, DNA binding and nucleic acid binding TF activity of the GO terms were significantly enriched (Additional file 18: Figure S5A). For the cellular components, the target genes are mainly found in CCAAT-binding factor complex, RNA polymerase II TF complex and nuclear TF complex (Additional file 18: Figure S5B). For the biological processes, target genes are significantly enriched into transcription (DNA-templated), nucleic acid-templated transcriptions, RNA biosynthetic process and so on (Additional file 18: Figure S5C). Furthermore, we performed KEGG pathway analysis on these target genes. The result showed that 87 miRNA target genes participated in 11 metabolic pathways, including environmental adaptation, lipid metabolism, nucleotide metabolism, etc. (Additional file 18: Figure S5D).

### qRT-PCR validation of miRNAs and their target genes

**Fig. 8:**
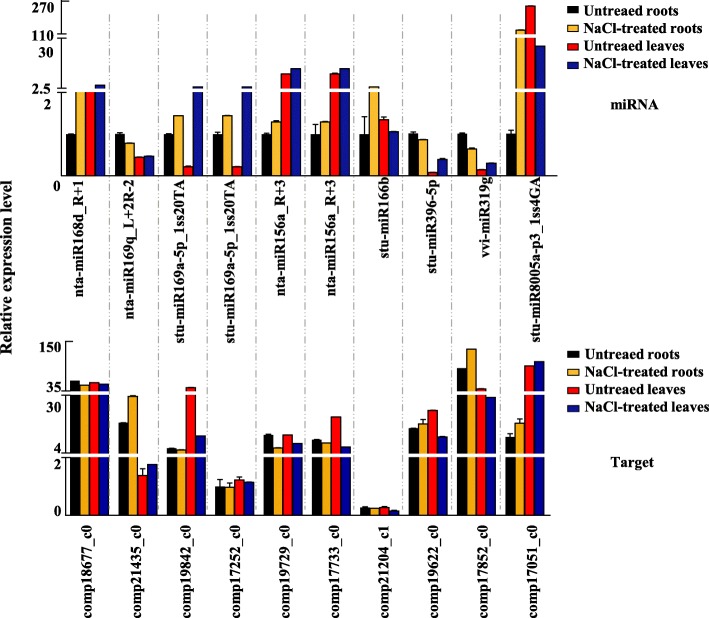
qRT-PCR analysis of miRNAs and their targets under salt stress. The relative expression of the 10 randomly selected sweet potato miRNA–target pairs were analysed by qRT-PCR. The ARF transcript levels were used for normalisation. Error bars indicate standard deviation. qRT-PCR value represents an average of three biological samples ± SE. MiRNA and its target primers are listed in Additional file 19: Table S14

To verify the small RNA sequencing results, we randomly selected 10 miRNA–target pairs for qRT-PCR analysis (Fig. 8). The results showed that in the leaves, the expression of eight miRNA–target pairs (nta-miR156a_R + 3/*SPL12*/*SL13A*, stu-miR169a-5p_1ss20TA/*NF-YA9*/*CRK1*, stu-miR166b/*ATHB-15*, nta-miR168d_R + 1/*AGO1*, stu-miR396-5p/*rpb-5*, stu-miR8005a-p3_1ss4GA/*EGD1* and vvi-miR319g/*TCP2*) displayed reverse expression pattern, indicating that miRNAs repressed their corresponding targets, and two miRNA–target pairs showed ‘unrelated miRNA–target pair in expression’. Similarly, in the roots, eight miRNA–target pairs showed a reverse expression pattern, and two miRNA–target pairs showed ‘unrelated miRNA–target pairs in expression’. Even previously studies described that miRNAs and targets might be involved in multiple expression patterns and their co-regulation can transcend the inverse of the expression [40, 41]. However, this ‘unrelated miRNA–target pairs in expression’ might be possibly caused by the false positive effect of the transcriptome itself, which was need to be further investigated carefully in the future.

## Discussion

### High-throughput sequencing and identification of miRNAs in sweet potato

According to miRNA prediction criteria, 650 miRNAs, including 475 known miRNAs and 175 novel miRNAs, were obtained from 12 small RNA libraries. The use of multiple sRNA libraries can improve the reliability of novel miRNA prediction and provide a solid basis for further in-depth miRNA study in sweet potato. In our study, most of the known miRNA are 21 nt in length, and the novel miRNAs are mainly 21 and 24 nt in length (Fig. 3 and Additional file 10: Figure S3), which is consistent with the report of sRNA in sweet potato [42]. And the sRNA length distribution pattern in sweet potato is very similar to the typical pattern of sRNAs reported in *Betula luminifera* [43]. The difference in the length of small RNAs may be related to the species and the enzymes that process it. For example, the length of the small RNAs in plants processed by DCL1 and DCL2 is 21 and 24 nt, respectively [44]. The 21 or 22 nt miRNAs tend to function as silencing complexes with AGO proteins and cleave the target genes [45], whereas 24 nt miRNAs mediate DNA methylation by binding to AGO4 [46]. Among known miRNAs, most of the first base of 21 and 22 nt miRNA was U (uracil) biased at the 5′-end, and the first base 24 nt miRNA was A (adenine) biased. For 175 novel miRNAs, the first base of the 5′-end of miRNA was A (adenine), accounting for 33.91%, and U (uracil), accounting for 35.63%. Some studies have found that uracil (U) at the 5′-end can facilitate the miRNA and AGOl protein binding. The first base adenine (A) at the 5′-end of 24 nt sRNA is beneficial for binding to AGO4, which preferentially binds to siRNA [47–49]. The previous studies revealed that when MFEI was greater than 0.85, the sequence most likely originated from miRNAs [50]. In our study, the minimal folding free energy index (MFEI) of novel pre-miRNAs ranged from 0.90 to 2.50 with an average of 1.29, which was higher than that of other types of RNAs, such as tRNAs (0.64), rRNAs (0.59) and mRNAs (0.65) [51].

### Analysis of differential miRNAs in response to salt stress in sweet potato

Identification of differentially expressed miRNAs will provide a better understanding of the post-transcriptional regulation in sweet potato under salt stress. A total of 148 miRNAs (101 known miRNAs belonged to 24 miRNA families, 47 novel miRNAs) were significantly up- or down-regulated by salt stress treatment in sweet potato roots or leaves (*P* < 0.05) (Additional file 12: Table S8 and Fig. 5b). In the roots, miR169_1, miR171_1 and miR818 were significantly down-regulated under salt stress, whereas miR166, miR156, miR167_1 and miR159 were up-regulated. In the leaves, miR2111, miR8005 and miR827_5 were significantly up-regulated, whereas miR399, miR167–1, miR166, miR172, miR396, miR408, miR477, miR444, miR482, miR530 and miR2118 were significantly down-regulated. In the roots and leaves, the miR168 family was significantly up-regulated, whereas the miR169_1 family was significantly down-regulated. These results implied that down- or up-regulated miRNA may play negative or positive roles in salt response in sweet potato. Some miRNA members belonging to the same family showed opposite expression pattern after salt stress treatment in sweet potato roots and leaves. For example, in the roots, stu-MIR530-p3_2ss8GA20TG of the miR530 family was down-regulated, whereas tcc-miR530a_R + 1_1ss12CT was up-regulated. In the leaves, nta-miR156a_R + 3 was down-regulated, and gma-miR156b_L + 2R-1 was up-regulated. This result indicates that miRNAs in the same family may play different roles in response to salt stress. *Arabidopsis* miRNA168 and maize miRNA168 were previously reported to be induced by salt stress [52, 53], and maize miR167 and *S. linnaeanum* miR399b were down-regulated [52, 54]. This finding indicated that miRNAs, which show similar expression patterns in different species, may have similar salt stress response mechanisms. Although a large number of salt-stress-response-related miRNAs are conserved in plants, some miRNAs have different regulatory patterns in different species. For example, miR396 was significantly up-regulated in *Arabidopsis* under salt stress [53] but down-regulated in maize and cotton [52, 55]. The expression of miR172 is down-regulated in maize [52] but up-regulated in *Arabidopsis* [53]. Therefore, this species-specific miRNA regulation mechanism makes it necessary to analyse the miRNAs of specific plant species under salt stress.

### Dissecting the roles of sweet potato miRNA in salinity response by combined analysis of sRNA and degradome sequencing

**Fig. 9:**
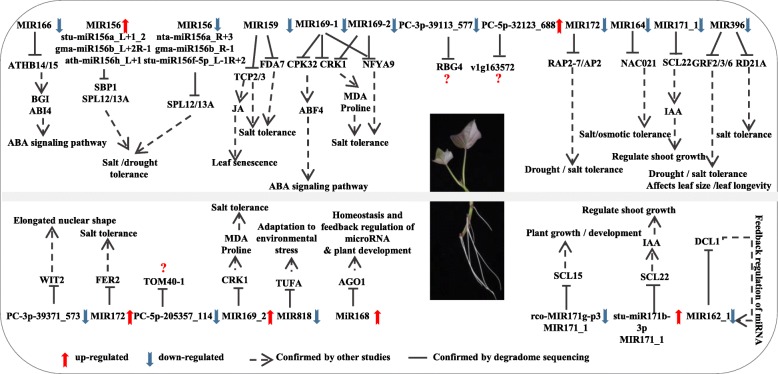
Potential regulatory network of miRNAs in the roots and leaves of sweet potato under salt stress

The target genes must be determined to elucidate the biological functions of miRNA. To further understand the regulation function of sweet potato miRNAs in salt stress response, we analysed the miRNAs and their target genes by miRNA expression profile, degradome and transcriptome. Sixty-six significant differentially expressed miRNAs and their 87 target genes were identified (Additional file 15: Table S11). These targets are TFs, hormone response genes, DNA/RNA binding proteins, protein-coding genes and enzymes. The general salt stress response pathway is: membrane receptors sense extracellular stress signals, signal transduction, transcriptional regulation and induction of salt-related gene expression and finally make physiological changes to cope with stress. Among them, salt stress signal transduction mainly includes ABA pathway, protein kinase pathway and SOS pathway. The potential regulatory network of miRNAs in sweet potato in salt stress response is shown in Fig. 9.

In sweet potato, nta-miR169q_L + 2R-2 targeted *CRK1* and *CPK32*, and nta-miR169q_R-2_1ss1CT and nta-miR169q targeted *CRK1*. The calmodulin-dependent protein kinases (CDPK) cascade pathway is an important signalling pathway in the protein kinase pathway. When plants receive salt stress signals, the Ca^2+^-dependent signal transduction pathway can transmit signals through the synergistic effect of Ca^2+^ concentration as a second messenger and CDPK to induce TFs to play a role in enhancing plant salt tolerance [56]. *AtCRK1* is involved in salt tolerance stress by altering the levels of MDA and proline in *Arabidopsis* [57]. CPK32 interacts with ABF4 and phosphorylates, and CPK32-overexpressing plants exhibit a highly sensitive phenotype for ABA and NaCl, suggesting that CPK32 is actively involved in ABA/stress signalling [58]. In the process of signal transduction, TFs receive upstream salt stress signals and regulate the expression of downstream salt-tolerant or salt-sensitive genes [59, 60]. In the present study, most of the targets of miRNAs were TF genes, including *NACs*, *SPLs*, *AP2*, *TCP* and *NF-Y*. *NACs* are the largest TF gene family in plants that play an important role in abiotic stress responses. OSNAC5 protein has been found to bind to the promoter region of *OsLEA3*, up-regulate the expression of stress-related genes and enhance rice stress tolerance [61]. In addition, *GmNAC021* is highly expressed in drought-resistant soybeans and may play a role in soybean leaf growth and plant response to drought stress [62]. We found that ptc-miR164a_R + 3 was significantly down-regulated under salt stress and targets *NAC021*, suggesting that the decrease in ptc-miR164a_R + 3 may release the expression of its target *NAC021* to regulate the salt stress adaption in sweet potato.

The highly conserved miR156/SPL module was also identified in sweet potato. For example, significantly down-regulated sweet potato nta-miR156a_R + 3 and gma-miR156b_R-1 targeted to *SPL12*, *SPL4* and *SPL13A*. qRT-PCR validation showed that nta-miR156a_R + 3/*SPL13A* target pairs presented a reverse expression pattern. Squamosa promoter binding protein-like (SPL) is a key regulator of plant abiotic stress tolerance. *VpSBP16* and *BpSPL9* enhance salt and drought stress tolerance in *Arabidopsis* and *Betula platyphylla*, respectively [63, 64], indicating that miR156 may also participate in the salt stress response in sweet potato. *AP2* TFs have been reported to be regulated by miRNA172 and play an important role in the transcriptional regulation of biological processes involved in the growth and development and in the response to abiotic stress [65, 66]. In our study, four miRNAs (stu-miR172a-3p_R + 1, stu-miR172a-3p, stu-miR172d-3p_1ss21GT, stu-miR172a-3p_R-1) targeted to *AP2*/*RAP2* were significantly down-regulated in leaves under salt stress, which may be responsible for the reduction of *AP2*/*RAP2* degradation in sweet potato under salt stress. Tobacco transgenic plant overexpressing chickpea *AP2* exhibited salt tolerance traits [67]. Overexpression of the sweet potato *RAP2–12* gene in *Arabidopsis* increased its tolerance to salt and drought [68].

MiRNA319 targeted to a plant-specific TF named *TCP*, which plays an important role in plant growth and development and in the response to biotic/abiotic stress [60, 69]. In the present study, significantly down-regulated Vvi-miR319g targeted to *TCP* (*TCP2* and *TCP3*) in sweet potato leaves under salt stress. qRT-PCR validation showed that vvi-miR319g/*TCP2* target pairs presented a reverse expression pattern. In switchgrass (*Panicum virgatum* L.), 29 in 42 TCP proteins were related to salt treatment [70]. miR319-targeted *TCP* plays a role in the response of plants to bentgrass under salt stress [71]. Nuclear factor Y (NF-Y) is a ubiquitous TF in almost all eukaryotic organisms and has a high affinity for the CCAAT cassette for transcriptional regulation of a large number of genes. Our study found that most of the miRNAs in the miR169 family, which were significantly down-regulated under salt stress, targeted to *NF-YA1*, *NF-YA2* and *NF-YA9* in sweet potato leaves. qRT-PCR validation showed that nta-miR169q_L + 2R-2/*NF-YA2* and stu-miR169a-5p_1ss20TA/*NF-YA9*/*NF-YA2* target pairs presented a reverse expression pattern. This result is consistent with the miR169 expression in *Spartina alterniflora* leaves under salt stress [72]. In *Arabidopsis*, most members in the *NF-Y* gene family are regulated by miR169, and the salt tolerance of *Arabidopsis* plant was enhanced in *PtNF-YA9*-overexpressing plants [73].

ATHB-14/15 are members of the HD-ZIP III TF family. This study found that stu-miR166b, which was significantly down-regulated by salt stress, targeted to *ATHB-14/15*. Previous study found that STTM165/166 triggers significant degradation of all endogenous miRNA165/166 members, resulting in the increased expression of its target gene *HD-ZIP III* (*PHB*, *PHV*, *REV*, *ATHB8* and *ATHB15*), which directly leads to sttm165/166 overexpression in *Arabidopsis* seedlings and salt tolerance enhancement [74]. At the same time, overexpression of sttm165/166 reduced the expression level of miR165/166d, disrupting miR165/166-mediated target inhibition, and the up-regulated expression of miR165/166 targets directly promoted the accumulation of ABI4 and BG1, thereby regulating ABA and the abiotic stress response and control of ABA homeostasis [75]. In the future, the target pair of stu-miR166b and ATHB-14/15 should be further studied to determine whether the salt stress signal is transmitted through the ABA signalling pathway in response of sweet potato to salt stress. Some other TF genes, such as *GRF* and *SCL*, were targeted by miRNA in sweet potato under salt stress. For instance, *GRF2*/*3*/*6* were targeted by miR396, which were significantly down-regulated; In *Arabidopsis*, up-regulation of *AtGRF1* and *AtGRF2* significantly increased leaf width and number of cotyledons, whereas overexpression of miR396a or miR396b resulted in the narrowing of transgenic plants [76]. Therefore, whether miR396 could increase leaf width and number of cotyledons of sweet potato must be investigated because the expression was up-regulated by salt stress. MiR171 cleaves scarecrow-like (*SCL*) genes, which is a subfamily in the GRAS family [77]. In rice, osa-miR171c is involved in the ABA-dependent pathway and affects stress-related gene expression, which contributes to salt stress tolerance [78]. In roots, both stu-miR171b-3p and rco-MIR171g-p3 were found to target the *SCL* genes, which may be involved in salt response in sweet potato.

In addition to TFs, many important enzyme and functional protein-encoding genes related to stress responses were also the targets of miRNA, such as *AGO*, *RKS1*, *FAD7*, *RD21A*, *FER2*, *TUFA* and *DCL1*. Sweet potato nta-miR168d_R-1 was up-regulated, and its target gene is the AGO1 protein, which is involved in miRNA biosynthesis. Stu-miR162a-3 targeting *DCL1* was also identified in sweet potato, and miR162 and miR168 are involved in the feedback regulation of miRNA biogenesis and functional pathways by regulating their target mRNAs (*DCL1* and *AGO1*) [79, 80]. In *Arabidopsis*, AGO1 is involved in miRNA-mediated guided mRNA cleavage process [79]. The increased expression of miR168 inhibits the synthesis of the target gene AGO protein to attenuate miRNA-mediated mRNA cleavage, resulting in increased levels of protein at the translational level, thereby activating and enhancing various physiological functional pathways of the plant [81]. *FDA7* was targeted by mes-MIR319e-p5_2ss12GC19GA targets in sweet potato, and antisense expression of *Arabidopsis FDA7* gene reduced salt tolerance in tobacco [82]. Significantly down-regulated sweet potato stu-miR396-5p_1ss21TG and osa-MIR818b-p3_2ss5TA18AC targeted to *RD21A* and *TUFA,* respectively, thereby playing an important role in plant adaptation to environmental stress [83]. The significantly up-regulated stu-mir172d-5p_1ss2gc of sweet potato targeted *FER2*, which played a very important role in salt stress resistance in cotton [84]. The function of these sweet potato miRNAs and their corresponding targets participating in environmental stress adaption must be studied further.

## Conclusions

In conclusion, through transcriptome, sRNA, and degradome sequencing, hundreds of known and novel miRNAs were identified, and their expression patterns were comprehensively analysed under salt stress treatment. We found that miRNAs specifically targeted many mRNAs, including TFs, signalling proteins, enzymes, etc., in sweet potato, thereby negatively regulating gene expression. This study is the first to demonstrate the miRNA-mediated gene regulation in sweet potato under salt stress, which will provide insights into the understanding of miRNA-mediated salt stress response regulatory networks in sweet potato and construct solid theoretical foundation for molecular breeding of sweet potato by miRNA manipulation.

## Methods

### Plant materials and salt treatments

The sweet potato cultivar Xu32 used here was same as our previous studies [85, 86], which was bred by Hongmin Li and deposited (deposition number: GPD ganshu 2018–320,002) in the Xuzhou Academy of Agricultural Sciences/Sweet Potato Research Institute, CAAS, Xuzhou, China. Tuberous roots of Xu32 were collected from CAAS and then grown in a greenhouse with a temperature range of 20 °C to 25 °C, a light intensity of 150 μmol/m^2^.s^1^ and a photoperiod of 16 h for 1 month. Afterwards, shoots with four to five functional leaves were cut from the tuberous roots and placed in a one-half strength Hoagland solution. After 10 days of culture, plants with fine roots and young leaves were subjected to salinity treatments (150 mM NaCl) for 2 days, whereas the plants in the control were not treated with NaCl. Three replicated samples for the roots and leaves were harvested into liquid nitrogen and stored at − 80 °C for total RNA extraction.

### RNA extraction, small RNA library construction and sequencing

Total RNA was extracted from SLC (Xu32 leaf without NaCl treatment as control), SLN (Xu32 leaf with NaCl treatment), SRC (Xu32 root without NaCl treatment as control), and SRN (Xu32 root with NaCl treatment) using TRIzol reagent (Invitrogen, CA, USA) according to the manufacturer’s procedure. Bioanalyzer 2100 and RNA 6000 Nano LabChip Kit (Agilent, CA USA) were used to analyse the quality and purity of total RNA. Twelve small RNA libraries were constructed following the standard procedures. The raw data have been uploaded into the NCBI database Sequence Read Archive (SRA) and the SRR numbers were from SRR10872161 to SRR10872172. Specifically, the 5′ and 3′ adaptor sequences were successively ligated to the aforementioned RNA fragments with T4 RNA ligase 2 [87]. The resulting RNA was reverse transcribed to cDNA and then subjected to 11 PCR cycles using the adaptor primers. The PCR product fragments with a of size 140–160 bp were isolated on 6% polyacrylamide Tris-borate-EDTA gel. RNA was then sequenced by Illumina HiSeq 2000/2500. In this study, RPKM (reads per kilobase of exon model per million mapped reads) was used to measure the abundance of gene expression. Gene differential expression analysis is the most noteworthy result in small RNA sequencing. |Log2foldchange| ≥ 1 and *P* value<0.05 was used to identify the differentially expressed genes. The Sweet potato Garden (http://sweetpotato-garden.kazusa.or.jp/) database was used as reference sequence database for transcriptome annotation.

### Identification of known and novel miRNAs

The raw data were processed with data cleaning analysis using ACGT101-miR v3.5 (LC Sciences, Huston, TX). In brief, the quality of raw data was measured by Illumina Fast QC to obtain Q30 data. Clean full-length reads were collected after removing all low-quality reads, adapter contaminants, and reads smaller than 18 nt and junk sequences (≥2 N, ≥7A, ≥8C, ≥6G, ≥7 T, ≥10 Dimer, ≥6 Trimer or ≥ 5 Tetramer). In addition, the clean data were filtered using various RNA databases, such as mRNA, RFam (release 9.1) and Repbase (version 15.07) databases, and rRNA, scRNA, snoRNA, snRNA, tRNA, etc. were found and removed as much as possible. The remaining unique sequences were mapped to the precursors in miRBase 21.0. by the fast gapped-read alignment software Bowtie 2 [88]. The unique sequences mapping to specific species mature miRNAs in hairpin arms were identified as known miRNAs. The unannotated sRNAs were expanded to about 250 nt and their structures were predicted using Mfold software (http://unafold.rna.albany.edu/?q=mfold). Novel miRNAs were obtained according to Meyers and Li prediction criteria [39, 89].

### Transcriptome and degradome sequencing and analysis

The total RNA was extracted from the four samples (SLC, SLN, SRC and SRN). Then the transcriptome cDNA library construction and sequencing methods are described previously [90]. Clean reads of four transcriptomes were obtained by filtering out adaptor sequences and low-quality reads of the original sequencing data, and then cleaned sequencing data were assembled using Trinity software. Finally, the gene obtained after assembly was compared with the protein sequences in the five public databases (Swiss-Prot, NR, KEGG, KOG and Pfam) (threshold value e ≤ 1e-10). Functional annotation was performed through sequence similarity. RNA of the NaCl-free (CK) and NaCl-treated (Na150) roots and leaves were prepared for degradome sequencing. Total RNA was extracted and captured by beads and connected with a 3′-5′ adaptor. Then, the whole library was constructed by using the mixed reverse transcription of Biotinylated Random Primers and mRNA and amplified by PCR. The constructed library was sequenced by Illumina HiSeq 2000/2500. Raw data obtained by sequencing were used to predict miRNA target genes using the CleaveLand 3.0 program [38] and ACGT301-DGEv1.0 program (LC Sciences, Houston, TX, USA). On the basis of the abundance of the resulting mRNA tags relative to the overall profile of the degradome reads that matched the target [91], all target genes were divided into five categories, namely, Category 0, 1, 2, 3 and 4. The depositing data numbers for the transcriptome and degradome were SRR10854671, SRR10873471 and SRR10873472 in NCBI database.

### qRT-PCR detection of expressed miRNAs and target genes

We performed expression verification of six differentially expressed miRNAs and eight target genes by qRT-PCR using TB Green™ Premix Ex Taq™ II (TaKaRa, Japan) and ABI plus sequence detection system (ABI, USA) as previous study [92]. Primer sequences for miRNAs and their targets are listed in Additional file 19: Table S14. Relative expression changes were calculated using the 2^−ΔΔCt^ method [93] with ADP-ribosylation factor (*ARF*) as an internal control gene. PCR reaction was performed with three biological replicates.

## Supplementary information

**Additional file 1: Table S1.** Summary of sequence data generated for sweet potato transcriptome and quality filtering.

**Additional file 2: Table S2.** Assembly statistics of reads.

**Additional file 3: Table S3.** Distribution of the assembled genes and transcript length.

**Additional file 4: Table S4.** Blast analysis of non-redundant unigenes against public databases.

**Additional file 5: Table S5.** Summary of sRNA sequencing data in sweet potato.

**Additional file 6: Table S6.** Identification of known miRNAs by high-throughput sequence.

**Additional file 7: Figure S1.** Distribution of conserved miRNAs in species.

**Additional file 8: Figure S2.** Quantitative distribution and expression level analysis of miRNA family in four libraries (SRC, SRN, SLC and SLN). (A) Analysis of expression levels between miRNA families. (B) Distribution of conserved miRNAs in the miRNA family of SRC, SRN, SLC and SLN libraries.

**Additional file 9: Table S7.** Identification of novel miRNAs by high-throughput sequence.

**Additional file 10: Figure S3.** Analyses of the length distribution and nucleotide base bias of novel miRNAs. (A) Size distribution of novel miRNA. (B) Percentage of the first nucleotide bias in novel miRNAs. (C) Novel miRNA nucleotide bias at first position.

**Additional file 11: Figure S4.** Predicted secondary structures of potential novel miRNAs from sweet potato. Sequences indicated in green correspond to predicted miRNA.

**Additional file 12: Table S8.** Summary of the comparison of known and novel miRNAs between the control and salt libraries in sweet potato.

**Additional file 13: Table S9.** Known and novel miRNA targets identified by degradome sequencing.

**Additional file 14: Table S10.** CleaveL and pipeline category of degradome.

**Additional file 15: Table S11.** Target prediction results of the differentially expressed miRNAs between sweet potato DSN and DSC degradome libraries. (XLS 30 kb)

**Additional file 16: Table S12.** Targets of salt-responsive differentially expressed miRNAs.

**Additional file 17: Table S13.** Information of the significantly enriched GO terms for differentially expressed miRNA target genes.

**Additional file 18: Figure S5.** Functional analysis of target genes of differentially expressed miRNAs. (A) Significant enrichment of target genes in cellular component classification. (B) Significant enrichment of target genes in molecular function classification. (C) Significant enrichment of target genes in biological process classification. (D) KEGG analysis of differentially expressed miRNA target genes in response to salt stress.

**Additional file 19: Table S14.** Primer sequences of miRNA and their targets used for qRT-PCR.

## Data Availability

The datasets generated and analysed during the current study are available in the NCBI database SRA repository, SRR10854671, SRR10873471, SRR10873472, and from SRR10872161 to SRR10872172.

## Abbreviations

AGO: Argonaute
AUX/IAA: Auxin/ indole-3-acetic acid
CT: Cycle threshold
GO: Gene ontology
MFE: Minimum free energy
MFEI: Minimum free energy index
miRNAs: microRNAs
ncRNA: Non-coding RNA
pre-miRNA: precursor miRNA
qRT-PCR: quantitative real-time PCR
RISC: RNA-induced silencing complex
rRNA: ribosomal RNA
snoRNA: small nucleolar RNA
snRNA: small nuclear RNA
TF: Transcription factor
tRNA: transfer RNA
