# Supplementary material for: High-throughput deep sequencing reveals the important role that microRNAs play in the salt response in sweet potato (Ipomoea batatas L.)
Source: BMC Genomics. 2020 Feb 17;21:164. doi: 10.1186/s12864-020-6567-3 (PMC7027035; doi:10.1186/s12864-020-6567-3)
Supplement: Supplementary file 8 — Additional file 8: Figure S2. Quantitative distribution and expression level analysis of miRNA family in four libraries (SRC, SRN, SLC and SLN). (A) Analysis of expression levels between miRNA families. (B) Distribution of conserved miRNAs in the miRNA family of SRC, SRN, SLC and SLN libraries. [file 12864_2020_6567_MOESM8_ESM.ppt]

## Slide 1
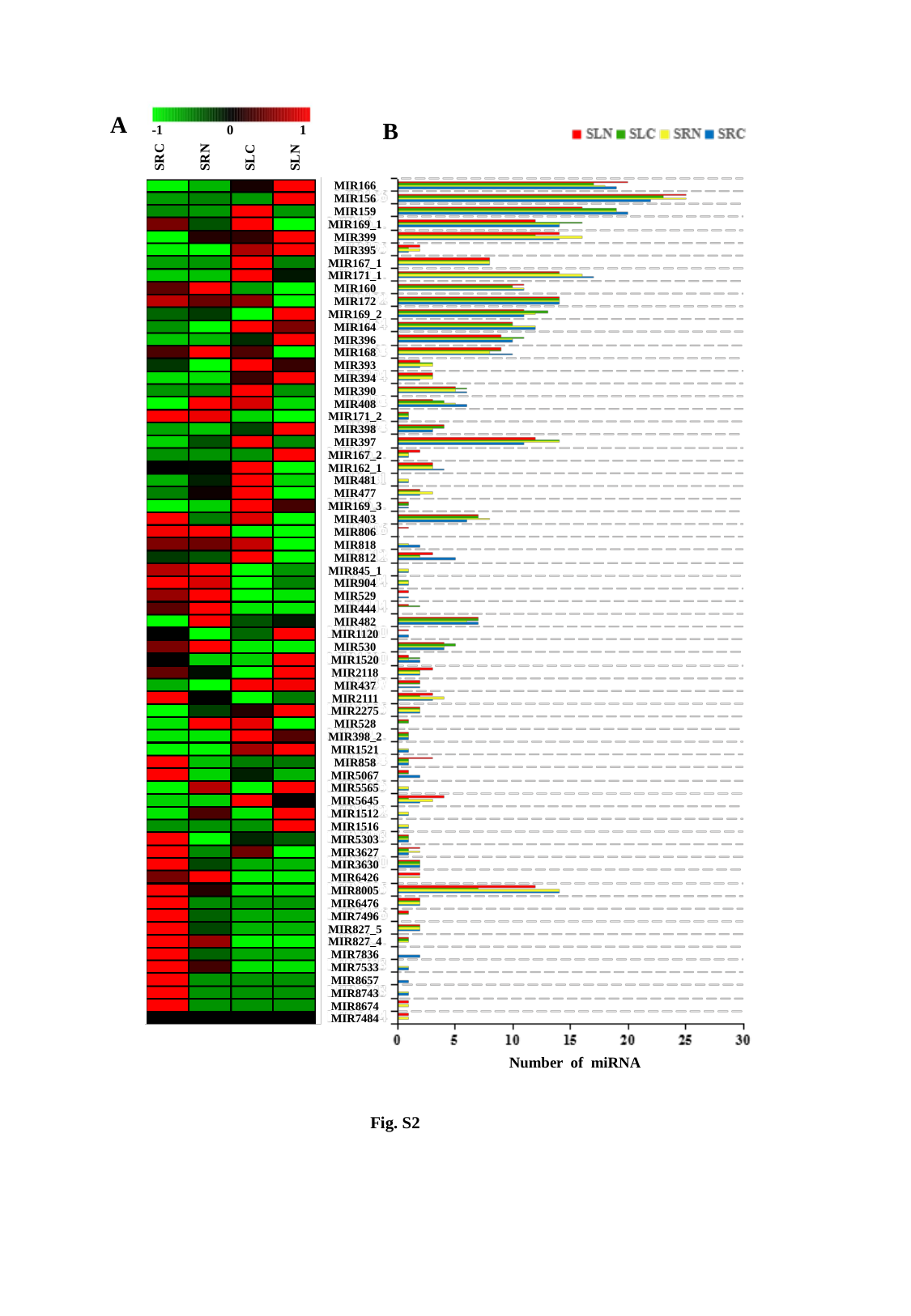

-1
0
1
A
SRC
SRN
SLC
SLN
B
MIR166
MIR156
MIR159
MIR169_1
MIR399
MIR395
MIR167_1
MIR171_1
MIR160
MIR172
MIR169_2
MIR164
MIR396
MIR168
MIR393
MIR394
MIR390
MIR408
MIR171_2
MIR398
MIR397
MIR167_2
MIR162_1
MIR481
MIR477
MIR169_3
MIR403
MIR806
MIR818
MIR812
MIR845_1
MIR904
MIR529
MIR444
MIR482
MIR1120
MIR530
MIR1520
MIR2118
MIR437
MIR2111
MIR2275
MIR528
MIR398_2
MIR1521
MIR858
MIR5067
MIR5565
MIR5645
MIR1512
MIR1516
MIR5303
MIR3627
MIR3630
MIR6426
MIR8005
MIR6476
MIR7496
MIR827_5
MIR827_4
MIR7836
MIR7533
MIR8657
MIR8743
MIR8674
MIR7484
Number of miRNA
Fig. S2
